# Molecular Profiles and Metastasis Markers in Chinese Patients with Gastric Carcinoma

**DOI:** 10.1038/s41598-019-50171-7

**Published:** 2019-09-30

**Authors:** Chao Chen, Chunmei Shi, Xiaochun Huang, Jianwei Zheng, Zhongyi Zhu, Qiaolian Li, Si Qiu, Zhiqing Huang, Zhenkun Zhuang, Riping Wu, Panhong Liu, Fan Wu, Shanyun Lin, Bo Li, Xiuqing Zhang, Qiang Chen

**Affiliations:** 1 BGI Education Center, University of Chinese Academy of Sciences, Shenzhen, 518083 China; 2 Fujian Medical University Union Hospital, Fuzhou, 350000 China; 3 BGI-Shenzhen, Shenzhen, 518083 China; 4 The Union Clinical Medical College of Fujian Medical University, Fuzhou, 350000 China; 5 China National GeneBank, BGI-Shenzhen, Shenzhen, 518120 China; 6 Fujian Provincial Key Laboratory of Translational Cancer Medicine, Fuzhou, 350000 China; 7 Fujian Medical University Stem Cell Research Institute, Fuzhou, 350000 China; 8 School of Biology and Biological Engineering, South China University of Technology, Guangzhou, 510006 China; 9 BGI-GenoImmune, Gaoxing road, East Lake New Technology Development Zone, Wuhan, 430079 China

**Keywords:** Cancer genomics, Metastasis

## Abstract

The goal of this work was to investigate the molecular profiles and metastasis markers in Chinese patients with gastric carcinoma (GC). In total, we performed whole exome sequencing (WES) on 74 GC patients with tumor and adjacent normal formalin-fixed, paraffin-embedded (FFPE) tissue samples. The mutation spectrum of these samples showed a high concordance with TCGA and other studies on GC. *PTPRT* is significantly associated with metastasis of GC, suggesting its predictive role in metastasis of GC. Patients carrying *BRCA2* mutations tend not to metastasize, which may be related to their sensitivity to chemotherapy. Mutations in *MACF1, CDC27, HMCN1, CDH1* and *PDZD2* were moderately enriched in peritoneal metastasis (PM) samples. Furthermore, we found two genomic regions (1p36.21 and Xq26.3) were associated with PM of GC, and patients with amplification of 1p36.21 and Xq26.3 have a worse prognosis (*P* = 0.002, 0.01, respectively). Our analysis provides GC patients with potential markers for single and combination therapies.

## Introduction

GC is one of the most common cancers and a leading cause of cancer death worldwide^1^, with a 5-year survival rate of about 30%^2^. The highest incidence is in East Asia, Central and Eastern Europe, and South Africa^3^. Surgery and chemotherapy are the mainstay treatments of GC, but nearly 20% GC patients develop peritoneal metastasis (PM), which is the most common form of metastasis of GC^4^. PM can lead to bowel obstruction and formation of massive amounts of malignant ascites, resulting in a poor prognosis^5–7^.

Several studies have used next generation sequencing strategies to determine the mutation spectrum of GC, and many significantly mutated driver genes have been identified, such as *TP53*, *ARID1A*, *PIK3CA*, and others^8–11^. However, most of these studies focus on the mutation profiles of GC and are mainly based on fresh frozen (FF) samples, but FF tissue has limited availability; therefore, little is known about the metastasis mechanism of GC, including peritoneal metastasis^12^. Formalin-fixing paraffin-embedding (FFPE) has been a standard sample preparation method for decades, and they are useful resources for cancer studies. There are many efforts to develop strategies to use FFPE specimens in cancer research, and several studies confirmed the technical feasibility^13–15^. However, these studies mainly use next-generation sequencing (NGS) target region panels, and WES has rarely been reported in studies with a large sample size.

Some factors are considered to be associated with the risk of PM, such as younger age, female gender, advanced T- and N-stage *et al*.^7^. Chemokines genes, such as *CXCL12* and *VEGF*, have been reported to be elevated in the development of PM^6^. Takeno *et al*. identified a 22-gene expression profile which is associated with PM^16^. Zhang *et al*. reported a case of GC with matched primary cancer and peritoneal metastatic tissue, identified several genes especially mutated in PM cancer^17^. These studies find some genes or clinical features which may plays a role in the prognosis of PM, but the molecular mechanisms by which GC undergoes PM are not completely elucidated yet.

In this study, we first performed WES on 74 FFPE samples of GC based on the BGIseq-500 platform, compared the molecular profiles of Chinese southern GC patients with TCGA and other cohorts, and then investigated the markers of metastasis of GC. We found that mutations in several genes and copy number variations (CNVs) of two genomic regions are associated with metastasis of GC, which can be further validated in large-scale studies.

## Results

### Patient characteristics

A total of 74 GC patients (hereinafter referred to as Fujian cohort) from Fujian Province, China, with complete clinical follow-up information were sequenced, including 32(43%) intestinal-type, 28(38%) diffuse-type,10(14%) mixed-type and 4(5%) indeterminate adenocarcinomas; 28 (38%) of the patients were less than 60 years of age, 46 (62%) were more than 60 years of age. The majority of the subjects were male (52,70%), and the remaining 22 (30%) were female. In all 7 (9%) were stage I, 8 (11%) stage II, 51 (68%) stage III, and 9 (12%) stage IV; and 57 (77%) patients had metastasis in a follow-up exam, of which 26 (35%) patients had peritoneal metastasis. The clinical characteristics and statistics were list in Table 1 and Supplementary Table S1.

**Table 1:** The clinical characteristics and statistics of GC cases included in this study (N = 74).

Characteristics	No. (%)	PM	Non-PM	*P* value^a^
**Age**				0.112
<60	28 (37.8)	13	15	
>=60	46 (62.2)	13	33	
**Sex**				0.885
Male	52 (70.3)	18	34	
Female	22 (29.7)	8	14	
**Lauren type**				0.232^b^
Intestinal	32 (43.2)	9	23	
Diffuse	28 (37.8)	12	16	
Mixed	10 (13.5)	3	7	
Indeterminate^c^	4 (5.4)	2	2	
**Tumor stage**				0.046^d,e^
Stage I	7 (9.5)	0	7	
Stage II	7 (9.5)	2	5	
Stage III	51 (68.9)	20	31	
Stage IV	9 (12.1)	4	5	
**Differentiation**				0.018
Poor	51 (68.9)	23	28	
Well	20 (27)	3	17	
Indeterminate^c^	3 (4.1)	0	3	

^a^Derived from a χ^2^ test unless otherwise specified. ^b^Intestinal-subtype versus diffuse-subtype. ^c^Indeterminate samples not include for difference tests ^d^stage II & III versus stage I. ^e^Derived from a Fisher’s exact test.

### Genomic mutations of Chinese GC patients

**Figure 1:**
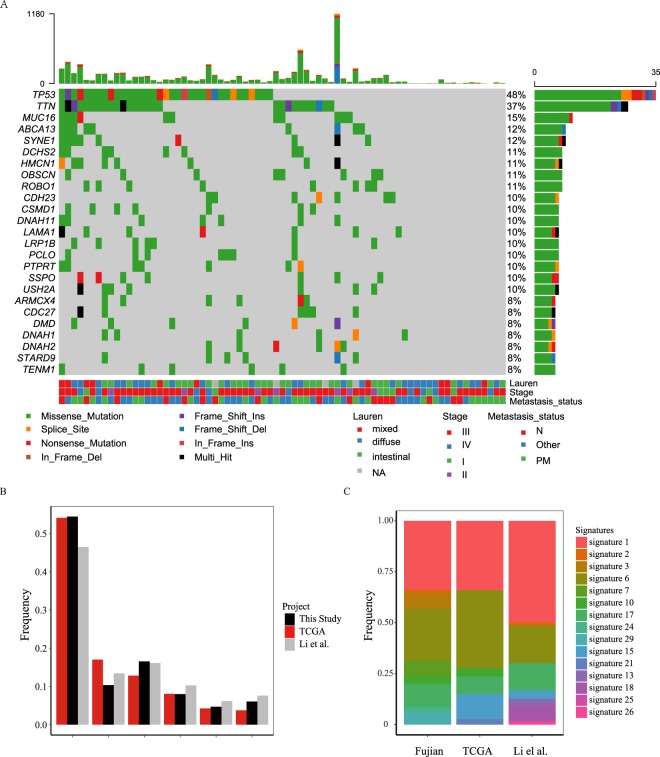
The mutation spectrum of GC in this study and the comparation with other studies. (**A**) Heat map showing somatic mutation profiles of cancer genes in this study. Left, the number of samples with mutations in a given gene. Top, the clinical type of samples and mutations burden of each sample. (**B**) The six classes of base substitution in three studies. (**C**) The mutation signatures in three studies.

A total of 11,118 mutations were detected in this study, the mean number of somatic mutations per patient was 150 (range from 0 to 1517) (Supplementary Table S2). Somatic SNVs (sSNVs) and indels (sIndels) accounted for 95.4% and 4.6% of the mutations, respectively. Of the mutations, 3,066 (27.6%) were synonymous, 6,857 (61.7%) missense, 463 (4.2%) nonsense (stopgain), 9 (0.1%) stoploss, 212 (1.9%) splice site, 452 (4.1%) were frameshift indels, and 59 (0.5%) were in-frame indels. Several cancer-related genes were frequently mutated in Fujian cohort, such as *TP53*(37/74)*, LRP1B*(8/74)*, PTPRT*(7/74), and *ARID1A*(5/74), consistent with previous studies on GC^10,11,18^ (Fig. 1A, Supplementary Table S3). Notably, all of the *ARID1A-*mutated samples carried wildtype *TP53* (*P* = 0.027), the mutation pattern had been reported in previous studies but was more pronounced in this study^8,9^. We randomly selected 36 mutation sites for mass spectrometry validation, and 34 (94.4%) of them were verified as somatic mutations (Supplementary Table S4).

The mutations in the exon and splice site regions of other two datasets, TCGA (download from https://cancergenome.nih.gov/) and Li *et al*.^18^ were used for further comparative analysis**(**Supplementary Table S1**)**. For point mutations, C > T, G > A transitions accounted for 54.4% of the sSNVs, and the ratio of the 6 types of base substitution is similar to the studies of TCGA and Li *et al*. (Fig. 1B). We further found that the spectrum of flanking nucleotides surrounding the mutated base was highly concordant between our results and the other two datasets (Supplementary Fig. S1A). The context-dependent mutational patterns of these three datasets were then identified using mSignatureDB (http://tardis.cgu.edu.tw/msignaturedb/) to explore the heterogeneity of mutagenic processes in GC and its diagnostic potential^19^. The results showed that prevalence of signatures 1, 6, and 17 were similar in the three studies, accounting for the majority of mutational processes (Fig. 1C). While signature 1 and 6 are related to spontaneous deamination of 5-methylcytosine and DNA mismatch repair, respectively, which results in C > T transitions and predominantly occurs at NpCpG trinucleotides^20,21^, other signatures specific to a study may be due to other endogenous mutational processes, treatment, or environment^22^.

We also found that the recurrently mutated genes in our study were similar to TCGA and Li *et al*., and the overlap between these three studies is about 50% **(**Supplementary Fig. S1B,C**)**. Some cancer-related genes that have been reported in other populations (Hong Kong and Russian) were also found frequently mutated in our cohorts, including *TP53*, *LRP1B*, *PTPRT*, *ARID1A*, *FAT4*, *FAT1*, and *APC*^10,11,18,23^. We found several genes especially mutated in each subtype of GC. *NUP214*(2/32, means 2 samples mutated among 32 samples, similarly hereinafter), *APC* (2/32), *PRDM16*(3/32), and *SMAD4* (2/32) altered more than once in intestinal-subtype tumors, but not in diffuse-type tumors. *DBX3*(2/28), *MYH9*(2/28), and *AFF3*(2/28) altered only in diffuse-type tumors (Fig. 1A). Notably, *RHOA* and *CDH1* are genes tend to frequently mutated in diffuse-type GC according to previous studies^10^, in this study, however, these two genes were mutated only once in diffuse-type tumors, while not in intestinal tumors.

Patients that were older (age >= 60) have significantly higher TMB (*P* = 0.0021) and TNB (*P* = 0.034) than younger patients (age < 60) (Supplementary Fig. S2A,B). and male patients tend to carry more mutations than female patients (P = 0.034), but the difference in TNB was not significant (*P* = 0.82) (Supplementary Fig. S2C,D). We didn’t find any significant difference of mutation burden between intestinal-type and diffuse-type tumors.

### Somatic copy number variations of Chinese GC patients

An analysis of copy number alterations of these 74 samples showed that most chromosome arms had undergone copy number gain or loss, with frequent amplified regions including 1q, 6p, 7, 8q, 13q, 20 (frequencies from 12% to 64%), and frequent losses observed on chromosomes 4, 14q, 18q, 19, 21q, 22q (frequencies from 16% to 43%) (Supplementary Fig. S3A). These overall somatic copy number variant (sCNV) patterns are consistent with previously published studies on GC^10,11,23,24^. We identified 156 focal amplifications and 69 focal deletions, in well-known oncogenes, such as *ERBB2*, *CCNE1*, *KRAS*, *MYC*, *EGFR*, and *CDK6*, and cancer-related genes such as *GATA4*, *GATA6*, *CD44* and *ZNF217* (Supplementary Table S5). Some tumor suppressor genes were identified in focal deleted regions, such as *CDKN2A*, *FAT1* and *SMAD4* (Supplementary Fig. S3B). These results are consistent with other studies such as TCGA and Wang *et al*.^10,11^. Through analyzing the sCNV at different subtypes of GC, we found that sCNVs occurred more frequently in intestinal-type and diffuse-type than mixed-type tumors (Supplementary Fig. S3C). Especially, there are 134 cancer-related genes amplified or deleted in intestinal-type tumors, much more than in diffuse-type (40) and mixed-type (4) tumors. Overall, we found 155 cancer genes amplified or deleted in our samples, in which half of them (78 genes) have been reported by TCGA or Wang *et al*. (Supplementary Fig. S3D, Table S6), the other half (77 cancer genes) with sCNVs identified in this study could be further confirmed for their involvement in the development of GC.

### Metastasis-associated driving genes in GC

**Figure 2:**
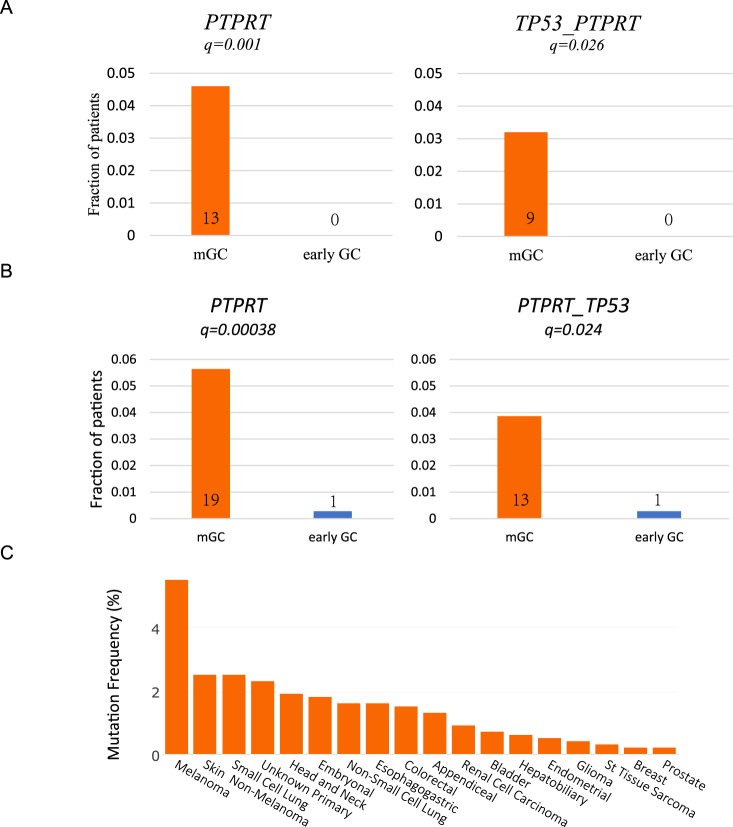
Association of mutations in *TP53* and *PTPRT* with GC metastasis. (**A**) Association of *TP53* and *PTPRT* gene mutations with metastasis in an independent cohort of 620 GC patients. (**B**) The correlation between *TP53* and *PTPRT* mutations and metastasis after integration of Fujian, MSK-impact and GENIE cohorts. (**C**) The frequency of *PTPRT* mutations in metastasis cancers in the MSK-impact pan-cancer study.

*PTPRT* has recently been reported to be closely related to early metastasis of colorectal cancer. In this study, we found *PTPRT* mutated in 7 patients (7/74), and metastasis occurred in six out of seven patients in clinical follow-up, suggesting that *PTPRT* may be associated with metastasis of GC. Because of the small number of samples in this study, we validated it with two published independent queues. We analyzed 620 GC patients from the MSK-Impact^25^ and GENIE^26^ prospective sequencing studies, including 280 patients with metastatic GC (Primary Stage IV or metastasis) and 340 patients with early GC (Primary, stage I-III). We then evaluated the relationship between the driving gene module and metastasis tendency in GC. It is noteworthy that, after correcting multiple hypothesis tests, we found that in this independent data set, the combination of *TP53* and *PTPRT* (q = 0.026), or *PTPRT* (q = 0.001) mutations alone were enriched in metastasis compared with early GC (Fig. 2A, Supplementary Table S7), similar to those reported by Zheng Hu *et al*. in colorectal cancer^27^. When the three cohorts were merged, the sample size increased to 694 (*PTPRT* mutation carriers increased to 20), and the correlation between mutation carriers and metastasis was more significant (Fig. 2B, q = 0.024 and q = 0.00038, respectively). We further analyzed the mutations of *PTPRT* in 10,000 metastatic cancers published by MSK-impact in 2017^28^.It was found that *PTPRT* has a considerable mutation frequency in many metastatic cancers, such as melanoma, small cell lung cancer, head and neck carcinoma, suggesting that *PTPRT* may be involved in multiple cancers (Fig. 2C).

In addition, we found that *BRCA2* mutation tended to enrich in non-metastatic samples (q = 0.02, Supplementary Table S7). Li *et al*. have reported that *BRCA2* mutation is associated with better prognosis of GC^18^. Evidence suggests that *BRCA2* mutation is related with improved platinum-based chemotherapy response and prolonged survival in patients with ovarian cancer^29^. It has been suggested that *BRCA2* mutation is associated with increased survival because of the reduced ability of *BRCA2* mutated cancer cells to repair damaged DNA caused by chemotherapy. Based on the above results, we speculate that GC patients with *BRCA2* mutation may benefit from chemotherapy to reduce the metastasis of tumors.

### Genomic alterations associated with PM

**Figure 3:**
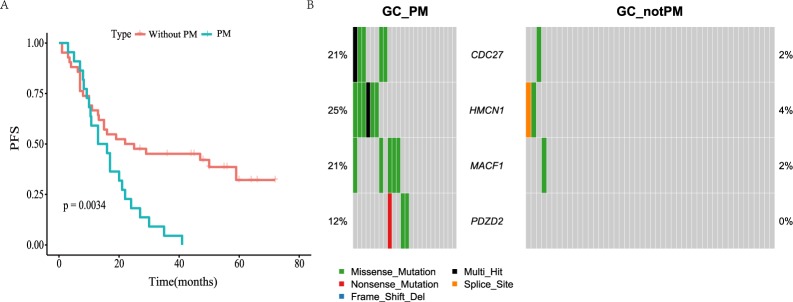
Genomic alterations and their prognostic significance associated with PM. (**A**) Kaplan-Meier plots for DFS in PM and not-PM patients. (**B**) Genes which enriched in PM patients. Fisher exact test, P < 0.05 and q < 0.1 (two GC samples without mutations were not considered in this analysis).

PM is the main form of metastasis of GC, and is an important cause of morbidity and mortality of GC patients. The patients with PM had a worse prognosis than those without PM in this study (*P* = 0.0034, Fig. 3A). In Fujian cohort, we found that the patients with advanced stage tend to occur PM (Stage II&III-VS-Stage I, *P* = 0.046), and poorly differentiated GC were more likely to develop PM **(**Table 1, *P* = 0.015**)**. In addition, we found that the younger patients (age < 60) tend to occur PM than older patients (age >= 60), although not statistically significant (*P* = 0.112).

To determine if there are mutation of genes specifically associated with PM, we compared the mutation pattern of genes between the PM group and Non-PM group, identified 3 genes (Fisher exact test, p < 0.05, q-value < 0.1), including *CDC27*, *MACF1* and *HMCN1*, which showed moderate enrichment in the PM group **(**Fig. 3B, Supplementary Table S8**)**. Among them, *MACF1* is a pan-cancer driver gene, which is related to cell adhesion function^4^. Interesting, we also found the gene *PDZD2*, which was previously supposed to involved in the early stages of prostate tumorigenesis, was especially altered both in this study (Fig. 3B, mutated in three PM sample) and the PM tissue of the GC patient in Zhang *et al*.’s study^17^. Similarly, we studied the relationship between PM and gene mutation in MSK-Impact cohort. There were 88 samples of PM in MSK-Impact cohort, and *CDH1* gene was enriched in PM samples (Supplementary Table S8). However, the MSK-Impact panel does not contain the four genes (*CDC27*, *MACF1*, *PDZD2* and *HMCN1*) mentioned above, so the relationship between the mutations of these genes and PM needs to be studied in a larger sample size.

Furthermore, we found that the amplification of several regions is enriched in PM patients, and two of them (1p36.21 and Xq26.3) are associated with a worse prognosis (Supplementary Fig. S4A,B). Interestingly, the 1p36.21 region contains a gene family named *PRAME* (preferentially expressed antigen of melanoma), which is expressed in many cancers and was functions in reproductive tissues during development^30^. *PRAME* had been reported as an independent marker for metastasis in uveal melanoma, indicating the value as a marker in the process of PM^31^. The Xq26.3 region contains a gene family named *CT45* (the Cancer/Testis Antigen Family 45), which is especially overexpressed in various cancer types. It had been reported that the family member *CT45A1* in breast cancer and lung cancer can act as a proto-oncogene to trigger tumorigenesis and cancer metastasis^32,33^. However, in the TCGA cohort, we did not find significant correlation between the amplification of these two regions and clinical prognosis. The reason may be the population difference between TCGA and Chinese, or the difference of other characteristics. Through the analysis of copy number alteration of PM samples, we found that there were significant deletions in many genomic regions of PM samples, involving many cancer genes and immune related genes (such as *B2M, RHOA, IFNE, JAK1/2*, etc.). KEGG pathway enrichment analysis showed that many cancer pathways, immune related pathways, such as JAK-STAT signaling pathway, Cell cycle, WNT signaling and Antigen processing and presentation pathway, were damaged (Table 2). However, this pattern is not obvious in non-PM samples.

**Table 2:** Enriched cancer-related pathway of peritoneal metastasis GC samples by GSEA analysis.

Gene Set Name (Pathway)	# Genes in Overlap (k)	# Genes in Gene Set (K)	q-value
Cytokine-cytokine receptor interaction	51	265	3.05E-17
JAK-STAT signaling pathway	37	155	8.49E-16
Regulation of autophagy	17	35	6.91E-13
Chemokine signaling pathway	34	189	3.97E-11
Natural killer cell mediated cytotoxicity	27	137	7.66E-10
Colorectal cancer	17	62	1.40E-08
Antigen processing and presentation	19	88	8.22E-08
Pathways in cancer	35	325	1.01E-05
Cell cycle	18	124	7.34E-05
MAPK signaling pathway	28	267	1.32E-04
Focal adhesion	23	199	1.53E-04
T cell receptor signaling pathway	14	108	1.33E-03
B cell receptor signaling pathway	11	75	1.91E-03
Cardiac muscle contraction	11	78	2.62E-03
P53 signaling pathway	10	68	3.16E-03
VEGF signaling pathway	10	76	6.52E-03
Cell adhesion molecules (CAMs)	14	133	7.56E-03
Non-small cell lung cancer	8	54	8.41E-03
WNT signaling pathway	15	150	8.41E-03

## Discussion

Overall, the GC mutation profiles of Fujian cohort are similar to those of TCGA and other cohorts. The feasibility of WES sequencing strategy based on FFPE and BGI-seq platform for cancer research was confirmed by comparative analysis of different queues.

Metastasis is a major cause of cancer-related death in cancer patients, but the molecular determinants of this process are largely unknown. We found several genes related to metastasis of GC, and *PTPRT* mutation can be used as a specific marker of metastasis. In addition, we found that patients with *BRCA2* mutation had fewer metastases, which may be related to the benefit of chemotherapy in patients with *BRCA2* mutation. In the Fujian cohort, two samples carried mutations in *BRCA2*. One of the patients with missense mutations (NO592779, well differentiated, who received chemotherapy after surgery) had no metastasis during clinical follow-up and had a progression-free survival of 26 months. However, another patient (NO573713, poorly differentiated) carried a missense mutation and a frameshift deletion of *BRCA2*, which progressed and metastasis 15 months after surgery. It is noteworthy that this patient’s mutation load is the highest in the Fujian cohort and carries a series of loss of function mutations of tumor suppressor genes (Supplementary Table S9 and 10).

PM can lead to bowel obstruction or malignant ascites, resulting in a poor prognosis and decline in the quality of life, so it is important to identify risk factors for PM. Mutations in *CDH1, MACF1, CDC27* and *HMCN1* were associated with PM, but this need to be confirmed in a larger cohort. We also found 2 regions, 1p36.21 and Xq26.3, that are amplified in PM patients, and associated with a poorer outcome. Importantly, two gene families of these amplified regions, *PRAME* and *CT45*, had been reported to overexpressed in various cancer types and associated with cancer metastasis^6,9,17,31^, indicating their potential as biomarkers for PM in GC patients. 9p21.3 contains several cancer genes and immune genes, which are significantly deleted in peritoneal metastasis samples. Previous studies have suggested that the absence of this region is associated with worse prognosis and limited benefit from immunotherapy^34,35^. On the other hand, at the CNV level of all cancer-related genes, we found that *ERBB2* (also known as HER2) amplified samples were more likely to have no peritoneal metastasis (q = 0.043, Supplementary Table S11), which might be a better treatment for HER2-amplified patients, such as HER2 inhibitors^25^. As the sample size of our study is limited, further studies should be conducted to confirm these findings.

## Materials and Methods

### Patient cohort

This study was approved by the Ethical Committee of the Union Medical College Hospital Affiliated of Fujian Medical University and carried out according to the approved guidelines. All patients signed informed consent prior to their enrollment. In total, 300 cases with sufficient clinical pathological information were provided;155 of which with pathological paraffin blocks were selected for WES. Samples of cancer and adjacent normal tissues were taken from each case at the same time, a total of 6 FFPE sections with size of 10 μm in 1 cm × 1 cm and tumor content of more than 50% were selected. Of the selected samples, 74 were successful for subsequent library construction and sequencing (Supplementary Table S1). GC data of MSK-impact (n = 204) and GENIE (n = 416) were downloaded from http://synapse.org/genie and cBioPortal (id = egc_msk_2017), respectively.

### WES library construction and next-generation sequencing

The genomic DNA of FFPE samples was randomly fragmented and the size of the library fragments was mainly distributed between 150 bp and 250 bp. The end repair of DNA fragments was performed, and an “A” base was added at the 3’-end of each strand. Adapters were then ligated to both ends of the end repaired dA tailed DNA fragments for amplification and sequencing. Size-selected DNA fragments were amplified by ligation-mediated PCR, purified, and whole-exome capture was performed using the BGI Human All Exon V4 kit. Captured products were then circularized. Rolling circle amplification (RCA) was performed to produce DNA Nanoballs (DNBs). Each resulting qualified captured library was then loaded on BGISEQ-500 platform and pair-end 50 bp or pair-end 100 bp sequencing was conducted for each captured library. We sequenced an average of 1,533,107,107 reads for each sample, after reads quality filtering and duplication removing, the sequencing depths for FFPE tumors and corresponding normal tissues were 117X and 92X on averages, respectively.

### Identification of somatic mutations

The sequencing data processing and variants detection pipeline is shown in Supplementary Fig. S5. Reads containing sequencing adapters and low-quality reads were removed using SOAPnuke software^36^. Then the high-quality data of each sample was mapped to the human HG19 reference genome and the duplicate reads were removed with Edico software (http://edicogenome.com/dragen-bioit-platform). To ensure accurate variant calling, local realignment around Indels and base quality score recalibration was performed using GATK^37,38^. Then the sequencing depth and coverage for each sample were calculated based on the alignments, and samples with low coverage or depth were re-sequenced on the same library to achieve enough sequencing depth.

SSNVs and sIndels were detected using the MuTect^39^ and Varscan2 software^40^, respectively. Then these mutations (sSNVs and sIndels) were annotated with ANNOVAR^41^ and followed by several filtering steps to remove potential false positives and obtain reliable results. For MuTect, in addition to the build-in filters, the following filtering criteria were applied: (1) total read count in tumor and normal DNA >= 10; (2) mutation allele fraction >=10% and >=5 reads that support this mutation; (3) mutation site is at least five bases away from the end of the read; (4) the SNV was not encompassed in short repeat regions; (5) presence of variant on both strands and the distribution of reads supporting this variant on the two strand is not biased; (6) the frequency of variant is less than 0.5% at 1,000 Genomes (1000G) database (http://www.1000genomes.org), Exome Sequencing Project (ESP) 6500 database (http://evs.gs.washington.edu/EVS) or Exome Aggregation Consortium (ExAC) database (http://exac.broadinstitute.org). For Varscan2, in addition to the built-in filters, the following filtering criteria were applied: (1) coverage >= 10 in normal DNA and coverage >=10 in tumor DNA; (2) variant frequency >=15%; (3) the Indel was not encompassed in short repeat regions; (4) the frequency of Indel is less than 0.5% at 1,000 Genomes (1000G) database, Exome Sequencing Project (ESP) 6500 database and Exome Aggregation Consortium (ExAC) database. The final mutation results were list in Supplementary Table S3.

SCNVs were detected by the CNV workflow tools within GATK4 (https://github.com/broadinstitute/gatk). The FFPE normal samples were used as control to identify tumor-specific genomic alterations. Then the copy-number segment data was used as input to the GISTIC2^42^ to detect recurrently amplified or deleted genomic regions. GSITIC2 analysis was performed using the default parameters.

### Confirmation of mutations

36 mutation sites, containing 21 cancer gene mutations and 15 mutations in PM samples specific genes were randomly selected for mass spectrometry validation. In total, 34 mutations were validated by the MassARRAY platform (including mutations that not been detected before, such as mutations in *NUP107*), with a 94% validation rate. We considered validation a success when both the tumor and normal genotype generated by MassARRAY platform were the same as the sequencing result, and failure if the genotype called by mass spectrometry was not the same as sequencing.

### Neoantigen prediction

SSNV mutations were used to predict neoantigens by NetMHC, NetMHCpan, PickPocket, PSSMHCpan and SMM^43^. The poor-quality peptides were removed according to two criteria: (1) IC50 < 500 in at least in three tools; (2) MT score < WT score for each peptide.

### Statistical methods

A Wilcoxon test was used to analyze the significance of the association of PM associated genes, patient age and patient gender. The Fisher exact test was used to analyze the significance of associations of the number of gene mutations with PM and not-PM. All tests were two-sided, and statistical significance was set at p < 0.05 or q < 0.05 if applicable. All statistical analyses were performed with RStudio software (Version 3.5.1)

## Supplementary information

Supplementary Dataset1

## Data Availability

The data reported in this study are available in the CNGB Nucleotide Sequence Archive (CNSA: https://db.cngb.org/cnsa; accession number CNP0000159).
