# Supplementary material for: Molecular Profiles and Metastasis Markers in Chinese Patients with Gastric Carcinoma
Source: Sci Rep. 2019 Sep 30;9:13995. doi: 10.1038/s41598-019-50171-7 (PMC6769015; doi:10.1038/s41598-019-50171-7)
Supplement: Supplementary file 1 — Supplementary Dataset1 [file 41598_2019_50171_MOESM1_ESM.pptx]

## Slide 1
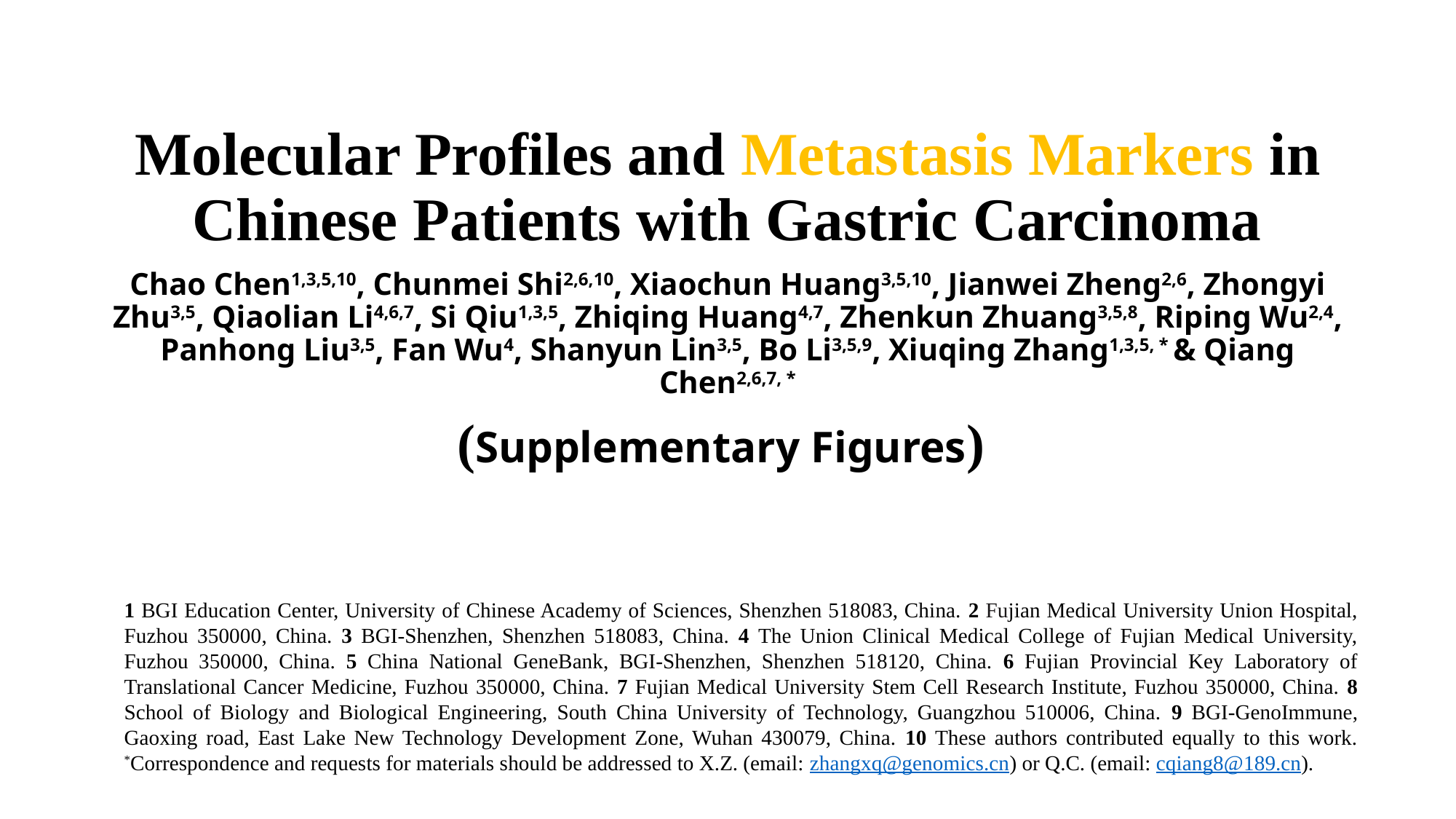

Molecular Profiles and Metastasis Markers in Chinese Patients with Gastric Carcinoma
Chao Chen1,3,5,10, Chunmei Shi2,6,10, Xiaochun Huang3,5,10, Jianwei Zheng2,6, Zhongyi Zhu3,5, Qiaolian Li4,6,7, Si Qiu1,3,5, Zhiqing Huang4,7, Zhenkun Zhuang3,5,8, Riping Wu2,4, Panhong Liu3,5, Fan Wu4, Shanyun Lin3,5, Bo Li3,5,9, Xiuqing Zhang1,3,5, * & Qiang Chen2,6,7, *
(Supplementary Figures)
1 BGI Education Center, University of Chinese Academy of Sciences, Shenzhen 518083, China. 2 Fujian Medical University Union Hospital, Fuzhou 350000, China. 3 BGI-Shenzhen, Shenzhen 518083, China. 4 The Union Clinical Medical College of Fujian Medical University, Fuzhou 350000, China. 5 China National GeneBank, BGI-Shenzhen, Shenzhen 518120, China. 6 Fujian Provincial Key Laboratory of Translational Cancer Medicine, Fuzhou 350000, China. 7 Fujian Medical University Stem Cell Research Institute, Fuzhou 350000, China. 8 School of Biology and Biological Engineering, South China University of Technology, Guangzhou 510006, China. ­9 BGI-GenoImmune, Gaoxing road, East Lake New Technology Development Zone, Wuhan 430079, China. 10 These authors contributed equally to this work. *Correspondence and requests for materials should be addressed to X.Z. (email: zhangxq@genomics.cn) or Q.C. (email: cqiang8@189.cn).

## Slide 2
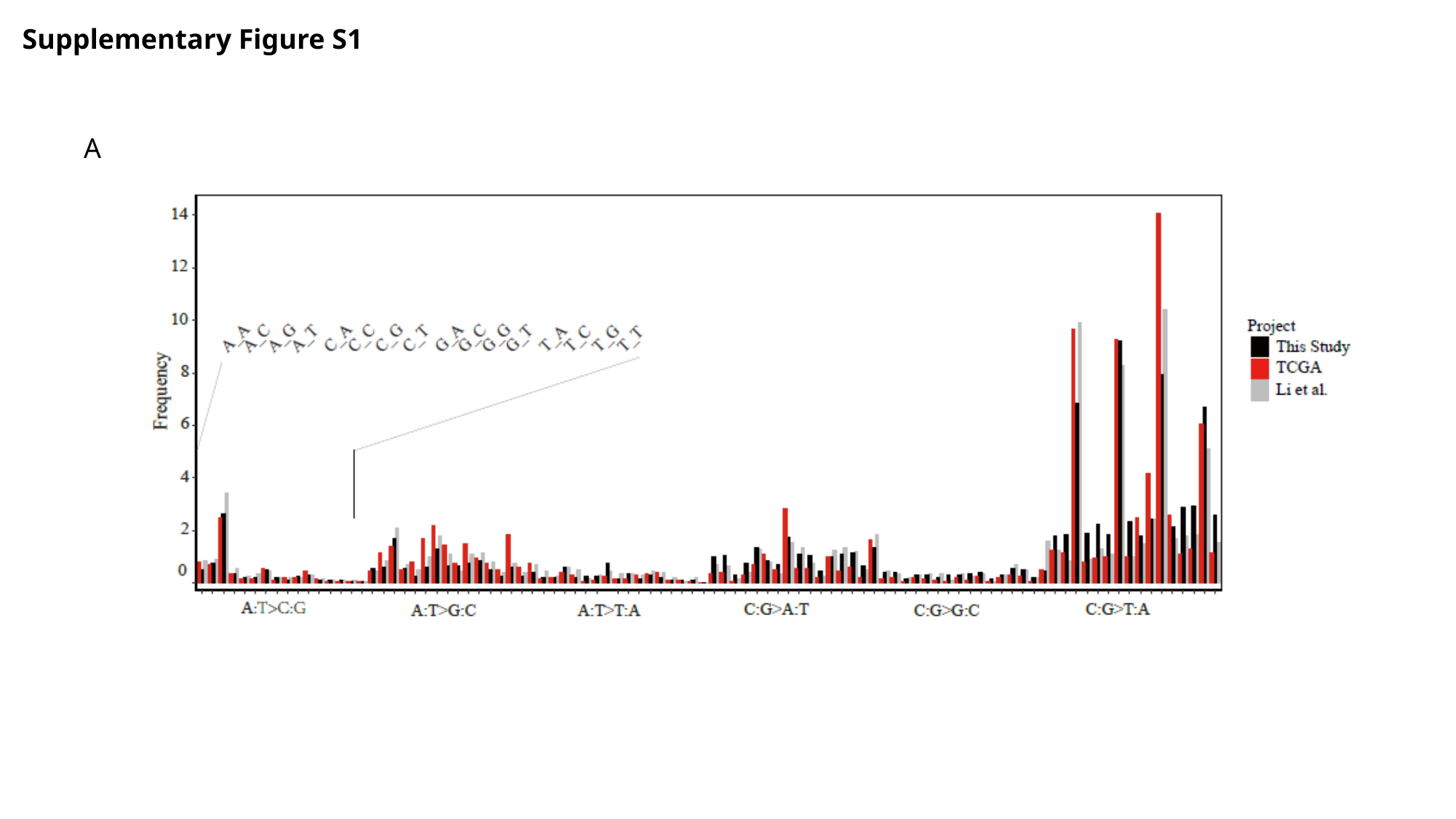

Supplementary Figure S1
A

## Slide 3
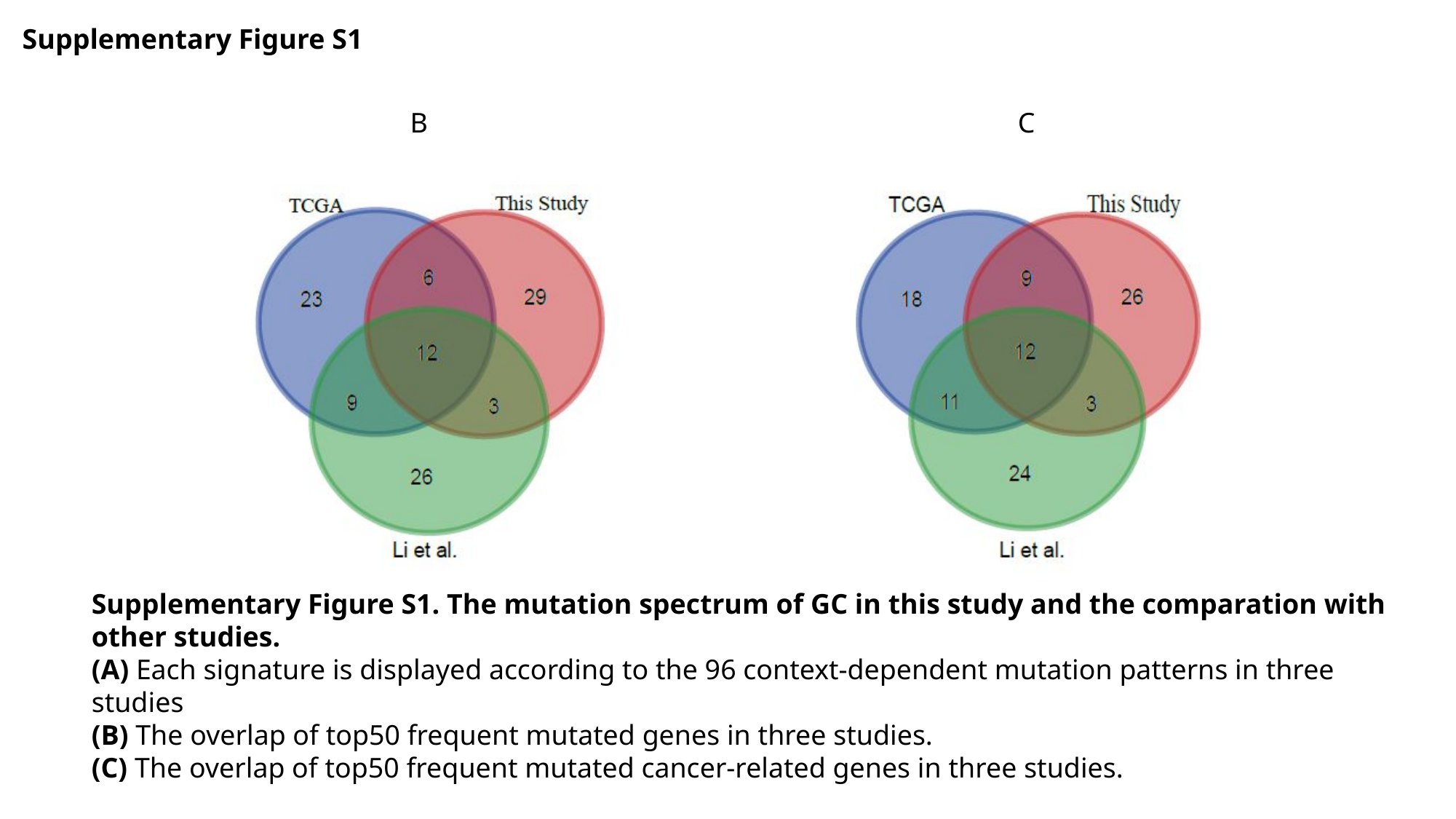

Supplementary Figure S1
B
C
Supplementary Figure S1. The mutation spectrum of GC in this study and the comparation with other studies.
(A) Each signature is displayed according to the 96 context-dependent mutation patterns in three studies
(B) The overlap of top50 frequent mutated genes in three studies.
(C) The overlap of top50 frequent mutated cancer-related genes in three studies.

## Slide 4
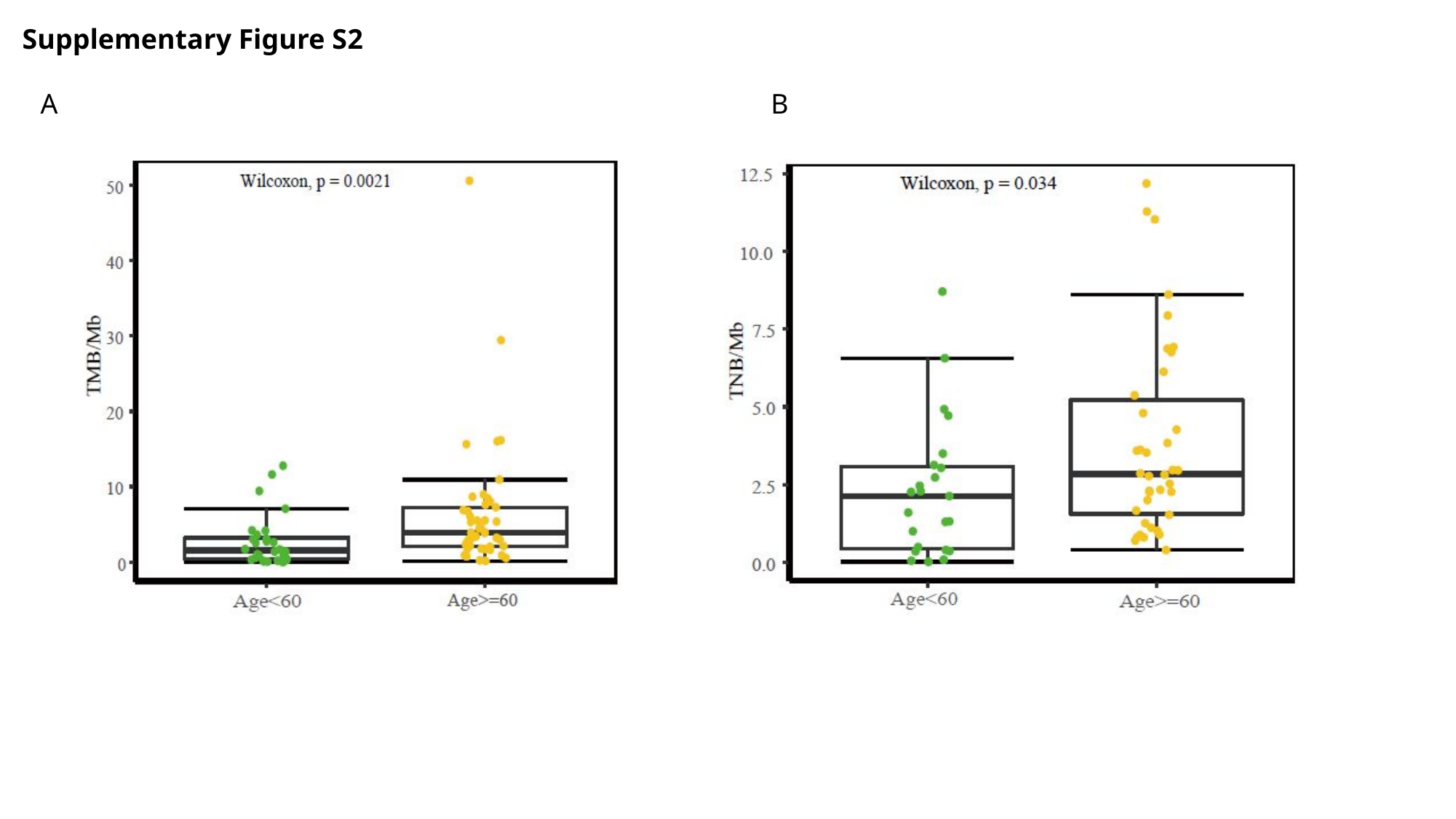

Supplementary Figure S2
A
B

## Slide 5
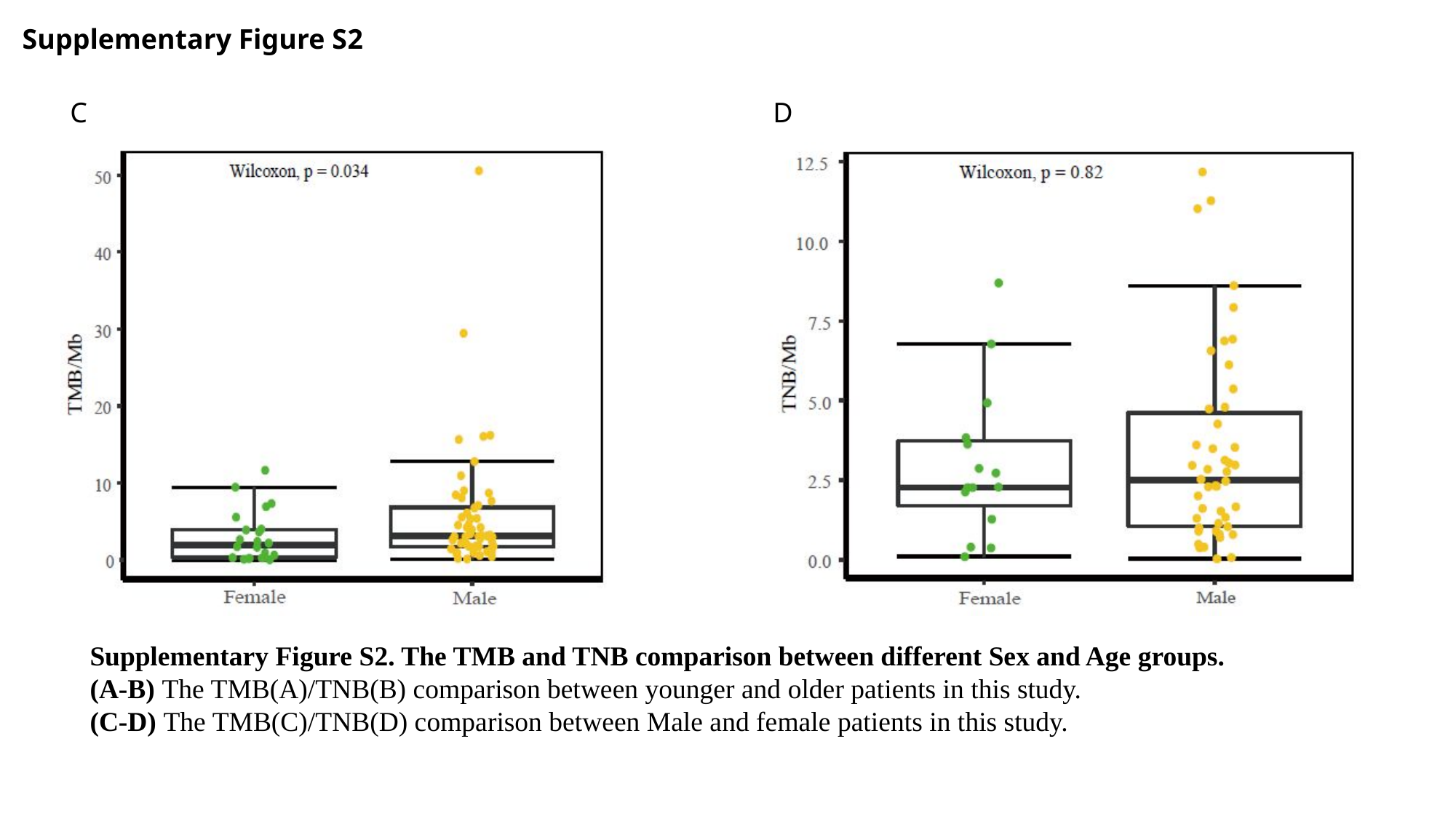

Supplementary Figure S2
D
C
Supplementary Figure S2. The TMB and TNB comparison between different Sex and Age groups.
(A-B) The TMB(A)/TNB(B) comparison between younger and older patients in this study.
(C-D) The TMB(C)/TNB(D) comparison between Male and female patients in this study.

## Slide 6
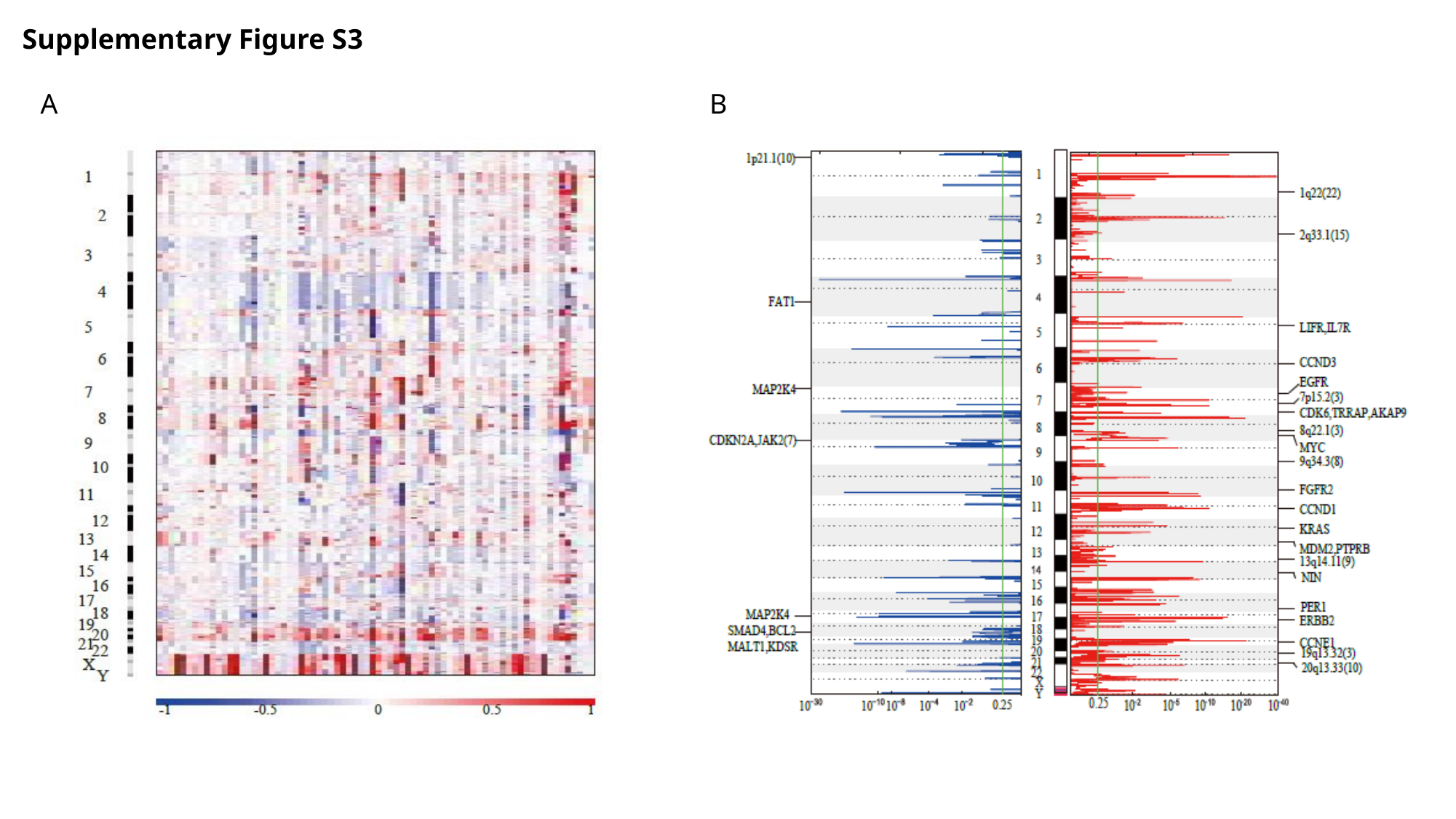

Supplementary Figure S3
A
B

## Slide 7
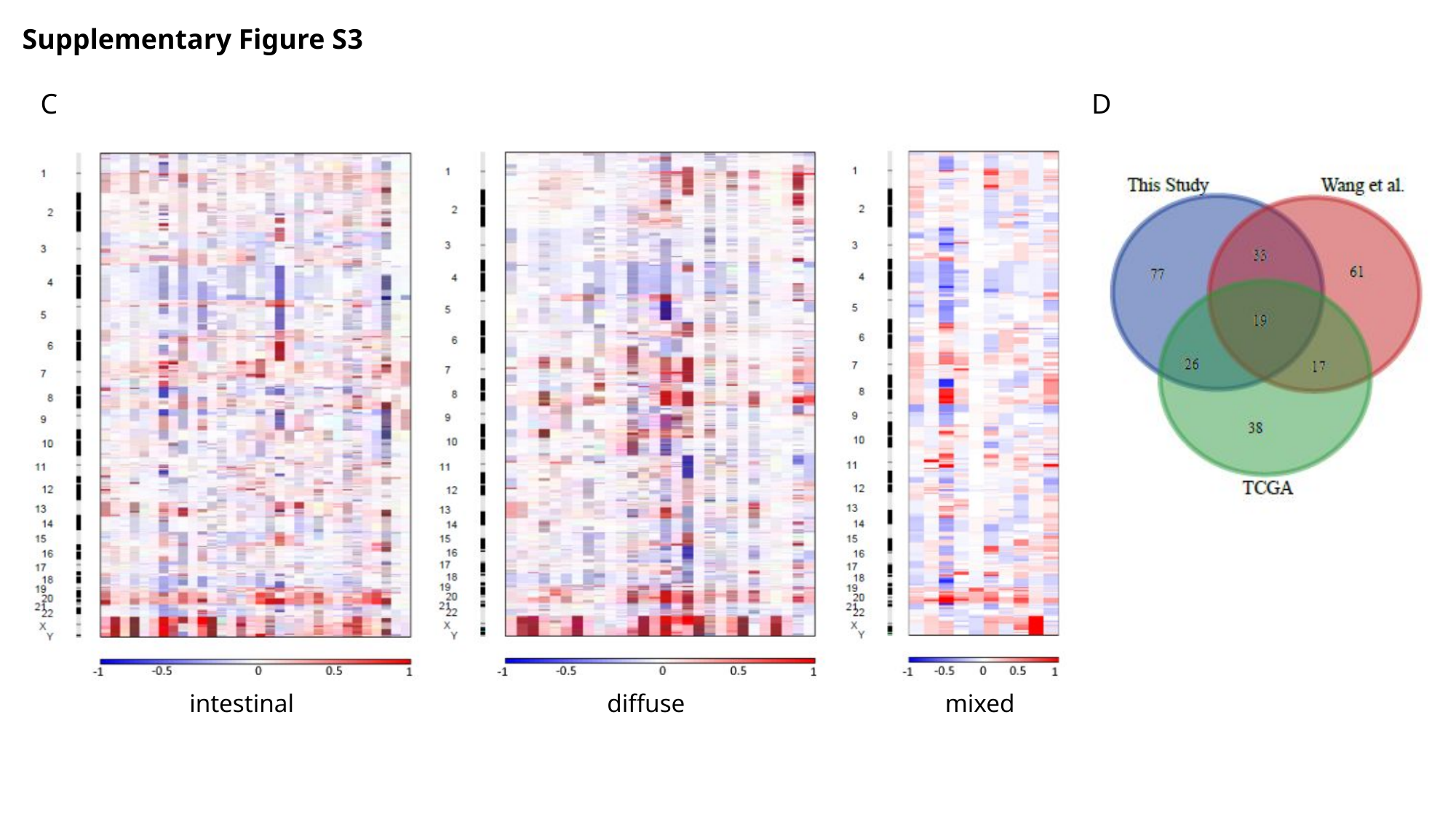

Supplementary Figure S3
C
D
intestinal
diffuse
mixed

## Slide 8
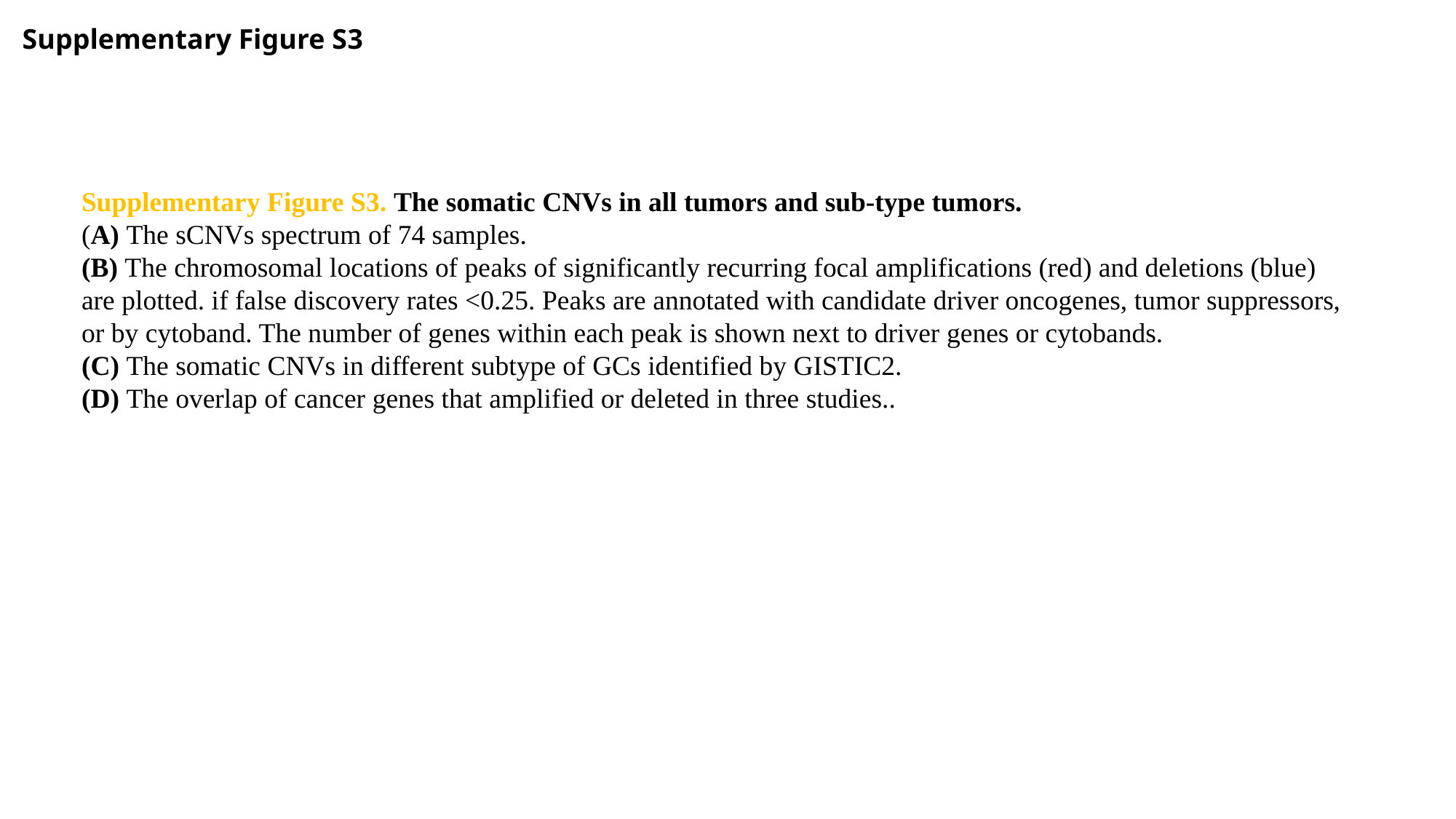

Supplementary Figure S3
Supplementary Figure S3. The somatic CNVs in all tumors and sub-type tumors.
(A) The sCNVs spectrum of 74 samples.
(B) The chromosomal locations of peaks of significantly recurring focal amplifications (red) and deletions (blue) are plotted. if false discovery rates <0.25. Peaks are annotated with candidate driver oncogenes, tumor suppressors, or by cytoband. The number of genes within each peak is shown next to driver genes or cytobands.
(C) The somatic CNVs in different subtype of GCs identified by GISTIC2.
(D) The overlap of cancer genes that amplified or deleted in three studies..

## Slide 9
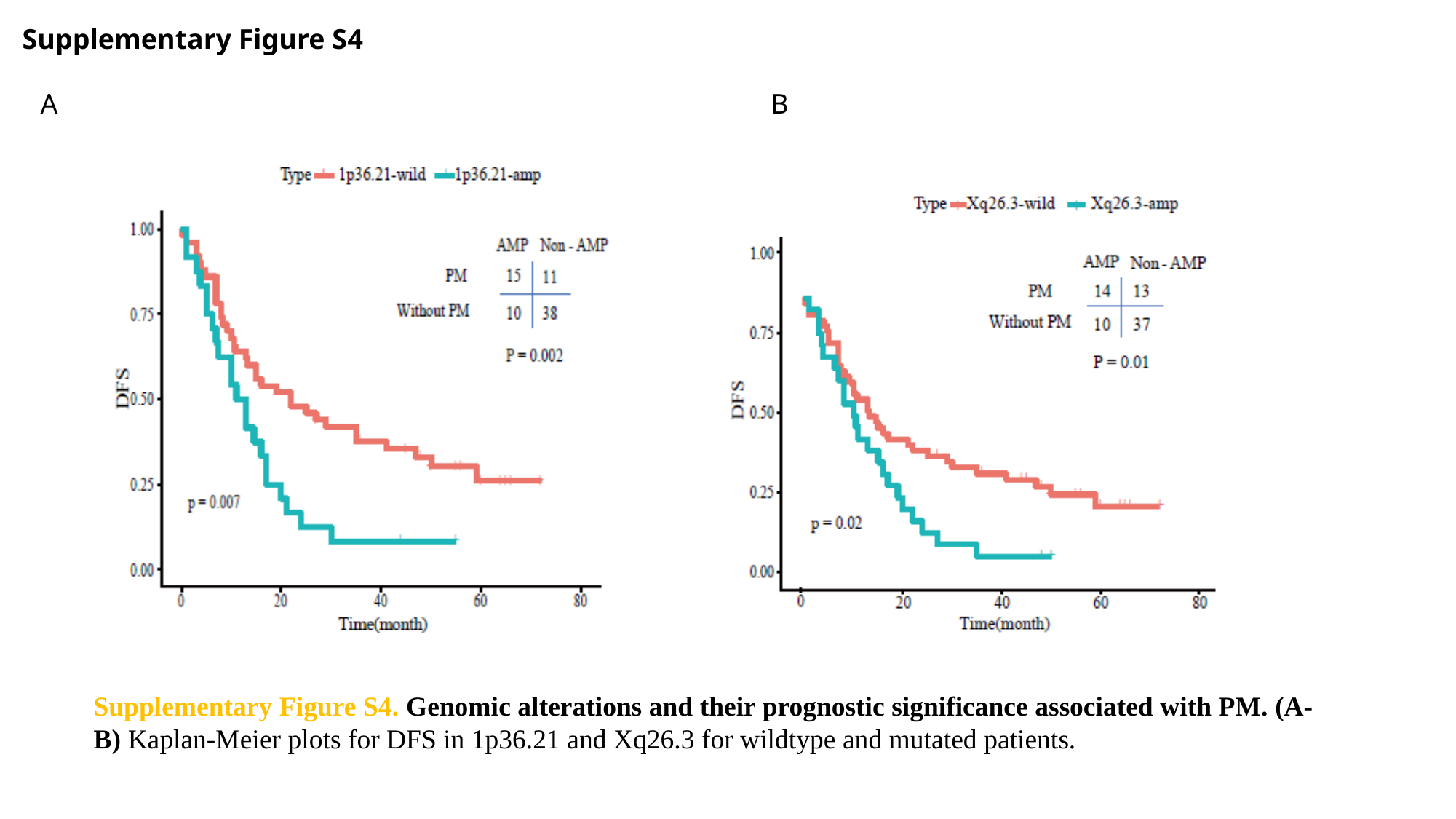

Supplementary Figure S4
A
B
Supplementary Figure S4. Genomic alterations and their prognostic significance associated with PM. (A-B) Kaplan-Meier plots for DFS in 1p36.21 and Xq26.3 for wildtype and mutated patients.

## Slide 10
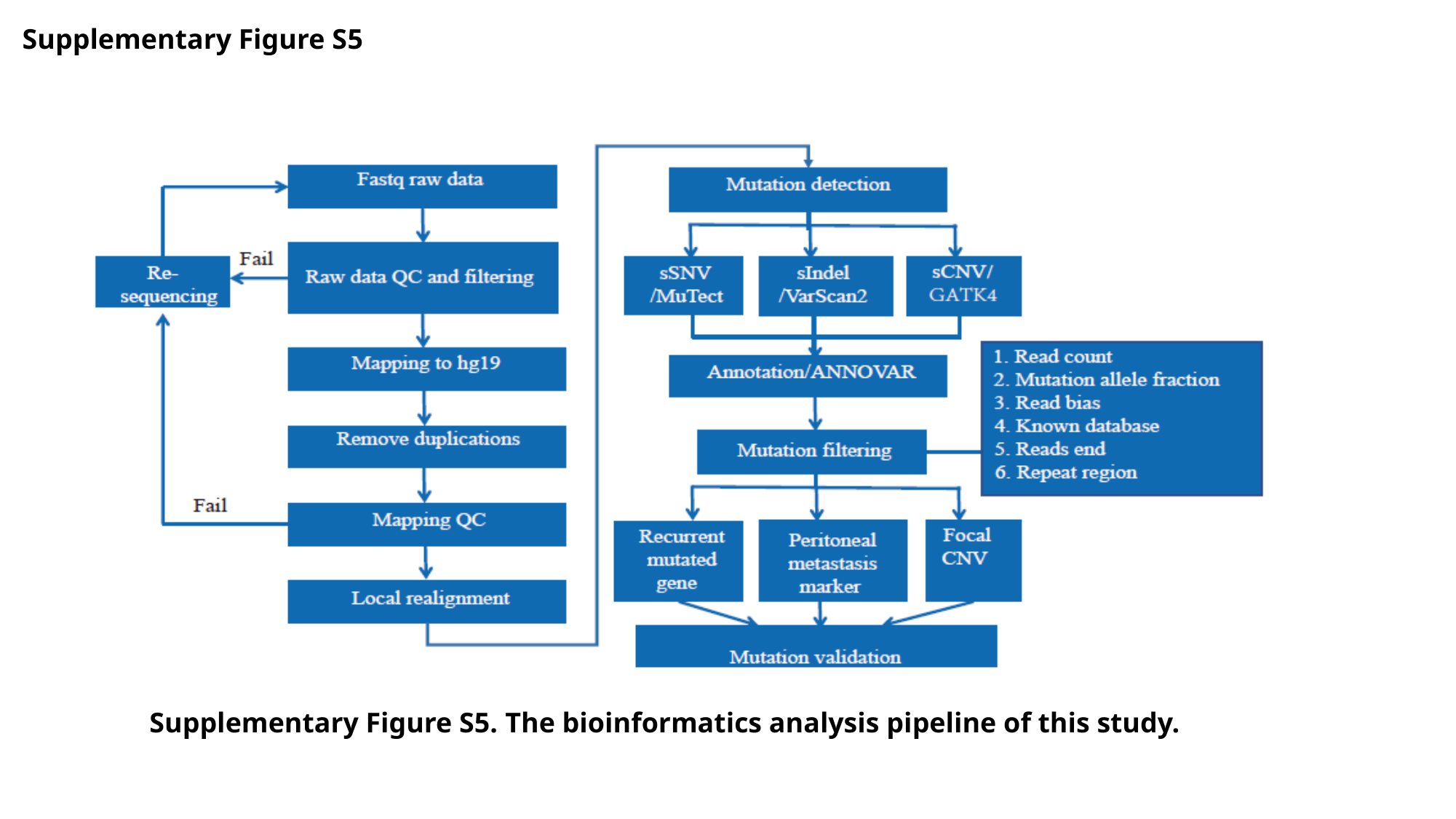

Supplementary Figure S5
Supplementary Figure S5. The bioinformatics analysis pipeline of this study.
